# Rush Medical College

**Published:** 1856-10

**Authors:** 


*Rush Medical College.*

Since the annual announcement of this Institution for 1856–'7 was printed, the following changes have been made, in consequence of Dr. E. Andrews declining longer to continue in the position of Lecturer on Comparative Anatomy and Demonstrator. Dr. J. H. Hollister, of this city, has been appointed Demonstrator of Anatomy, and Dr. J. C. Morfit, Prosector to the Chair of Surgery. Both these gentlemen are well known and highly esteemed, and are eminently qualified for the respective stations to which they have been appointed.

